# Supplementary material for: Magnetoactive bistable soft actuators for programmable large shape transformations at low magnetic fields
Source: Nat Commun. 2025 Nov 5;16:9714. doi: 10.1038/s41467-025-64855-4 (PMC12589526; doi:10.1038/s41467-025-64855-4)
Supplement: Supplementary file 1 — Supplementary Information [file 41467_2025_64855_MOESM1_ESM.pdf]

Supplementary Information for

Magnetoactive Bistable Soft Actuators for Programmable Large Shape Transformations at Low Magnetic Fields

Hao Wen[1,2], Zihao Shao[1,2], Yuxuan Sun[1,2], Chiyuan Ma[1,2], Feihong Xiang[1,2], Liangyu Xia[1,2], Xinhui Zhu[1,2], Xiaoxiang Li[1,2], Liang Li[1,2,], Quanliang Cao[1,2,]**

[1] Wuhan National High Magnetic Field Center, Huazhong University of Science and Technology, Wuhan 430074, China.

[2] School of Electrical and Electronic Engineering, Huazhong University of Science and Technology, Wuhan 430074, China.

*Corresponding author, **Email**: quanliangcao@hust.edu.cn (Quanliang Cao), Liangli44@hust.edu.cn (Liang Li)

The PDF file includes:

Note S1. Definition of large deformation and low magnetic field strength in this work.

Note S2. Bistable boundary model and experimental investigation.

Note S3. Equivalent current (magnetic field) calculation model and energy consumption for the energy-saving waveform.

Note S4. Influence of gripper tentacle on gripping effectiveness.

Fig. S1. Schematic structure of a dome-shaped shell.

Fig. S2. Deformation processes of monostable and bistable shells.

Fig. S3. Bistable dynamic process showing the transition from State B to State A.

Fig. S4. Bistable dynamic process showing the transition from State A to State B.

Fig. S5. Magnetization of circular magnetic soft samples by the hemispherical shell mold method.

Fig. S6. Comparison of shape deformation curves under different actuation waveforms.

Fig. S7. Opening characteristics of the magnetic valve.

Fig. S8. Liquid pumping process under a 10 Hz, 20 mT sinusoidal magnetic field.

Fig. S9. Programming structure and display of the proposed metamaterial.

Fig. S10. Physical diagram and control strategy of the magnetic actuation system for reprogrammable metamaterials.

Fig. S11. Dimensions of the biomimetic sea anemone gripper.

Fig. S12. Effects of tentacle length and stiffness on the grasping efficiency of the sea anemone-inspired gripper.

Fig. S13. Principle of the self-recognizing grasping anemone gripper.

Fig. S14. Comparison of deformation processes of hemispherical shells with different sizes.

Table S1. Comparison of shape transformation characteristics of magnetoactive soft actuators.

Table S2. Performance comparison of magnetoactive soft diaphragm pumps.

Table S3. Comparison of bistable dome-shaped soft actuators.

Supplementary References

Supplementary Notes:

Note S1. Definition of large deformation and low magnetic field strength in this work

In principle, both "large deformation" and "low magnetic field strength" are relative terms without universally strict thresholds. In particular, the definition of a low magnetic field can vary significantly depending on material properties, actuator design, and application scenarios. Different studies may adopt different ranges based on their performance requirements and experimental setups. In this work, we offer working definitions for both terms based on commonly reported parameter ranges in the literature and the practical challenges associated with achieving such parameter values (See Table S1).

- **Large deformation:** We define large deformation as a shape change ratio larger than 0.5, where the shape change ratio is calculated as the ratio of the actuator's maximum deformation to a characteristic structural dimension (e.g., diameter or length). This threshold is meaningful because many existing magnetoactive soft actuators in the literature do not achieve this level, as shown in Table S1, which compares the shape change performance of magnetoactive soft actuators under fully edge-constrained and zero pressure difference conditions.
- **Low magnetic field strength:** We define low magnetic field strength as $B < 100$ mT, which aligns with several prior works in the field of magnetic actuation where driving fields below 100 mT are explicitly described as "low" or "weak" or "small"[1-3]. Additionally, as shown in Table S1, a significant number of reported soft actuators operate under magnetic fields exceeding this threshold, further supporting the validity of our classification in the research scope of this work.

Note S2. Bistable boundary model and experimental investigation

The structure of dome-shaped shell is schematically shown in Fig. S1. In this section, we analyze the relationship between the bistable critical criteria of such spherical shells and the geometric parameters of the shell. These parameters are considered the core factors influencing the bistable characteristics of shells [4]. The subsequent analysis begins with the eversion and inversion processes of the shell, which can be quantitatively described using the stretching strain energy density U_s and the bending strain energy density U_b. According to the theory of shell bending [5], these two energy densities are expressed as $U_s \sim Eh\alpha^4$ and $U_b \sim D(1/R)^2$, respectively, in which E represents the Young's modulus of the material, h represents the thickness of the shell, α represents the angle of a quarter of the shell, D represents the bending stiffness of the shell. The bending stiffness D can be described by

$$D = \frac{Eh^3}{12(1-\nu^2)} \tag{1}$$

where ν represents the Poisson's ratio of the material. Then the ratio of the stretching to the bending strain energy density can be expressed as

$$U_S / U_b \sim \frac{Eh\alpha^4}{D/R^2} = 12(1-\nu^2)\frac{R^2}{h^2}\alpha^4 \tag{2}$$

This ratio measures the ease of the stretching and bending processes of the shell, and its fourth root is usually adopted to investigate the deformation characteristics of the shell [6], described by

$$\lambda_d = \left[12\left(1-\nu^2\right)\right]^{1/4}\sqrt{\frac{R}{h}}\alpha \tag{3}$$

When the parameter λ_d changes, the deformation process of the shell exhibits monostable or bistable characteristics. There is a threshold for this parameter between the monostable and bistable states. Typically, the threshold does not significantly change with the shell angle α, and the prediction results from shallow shell bistability theory are highly accurate for $\alpha \to \pi/2$ [7]. Therefore, we use the following equation to approximately predict the transition from bistability to monostability in our hemispherical shell, which is previously obtained for analysis of shallow shell in the work reported by Taffetani et al. [7],

$$\lambda_d \geq 1.44\nu + 5.06 \tag{4}$$

For the silicone elastomers used in this work, they can typically be regarded as

completely incompressible materials [8]. Therefore, we take a Poisson's ratio of 0.5 as a reference value to study the bistable characteristics of the hemispherical shell. By combining equations (3) and (4) and substituting the Poisson's ratio value, the bistable boundary model of our hemispherical shell ($\alpha = \pi/2$) can be obtained ,

$$h \leq 0.221R \tag{5}$$

To further illustrate the effect of shell thickness on its bistable properties, we present the experimental results shown in Fig. S2. Under an applied downward magnetic field of 65 mT, shells of varying thicknesses undergo deformation between the upward-convex (State A) and downward-concave (State B) configurations. In Fig. S2(a), for the shell with a thickness of h = 1.5 mm, the shell started to return to State A upon removal of the magnetic field at t = 7.6 s and failed to remain in State B, reverting to State A by t = 15.9 s, thus demonstrating monostable behavior. Conversely, Fig. S2(b) shows that the thinner shell (h = 0.5 mm) exhibits a different outcome: upon removal of the magnetic field at t = 12.5 s, the shell remained stable in State B throughout the observation period from t = 12 to 40 s), confirming its bistable characteristics (Supplementary Movie 1).

Furthermore, we experimentally demonstrate that, under bistable conditions, both concave-up and convex-down shapes can maintain their stability in the absence of an external magnetic field. Taking a magnetic shell with R = 10 mm, h = 0.5 mm, and 50 wt% magnetic powder as an example, we set the initial state to state B (Fig. S3). From 0 to 7 s, the shell remained stable in state B without an external magnetic field. At t = 7 s, we applied a 20 mT upward vertical magnetic field by powering a DC current source (current set to 3 A). The shell began transitioning from state B to state A. By t = 9.7 s, the shell had fully switched to state A. At t = 15.9 s, the DC power was turned off, and the shell remained stable in state A without a magnetic field from t = 15.9 to 25.4 s. Similarly, we set the shell's initial state to state A, reversed the connection of the DC source and coil to apply a downward magnetic field, and repeated the process. The results are shown in Fig. S4. These two sets of experiments clearly demonstrate that the shell can remain stable in both state A and state B without an external magnetic field, and dynamic switching between the two states is achieved by controlling the applied magnetic field.

Note S3. Equivalent current (magnetic field) calculation model and energy consumption for the energy-saving waveform

For a standard sine wave current, the energy consumption in a single cycle is

$$i^2RT = I^2{}_0R\int_0^T \sin^2 wtdt = \frac{I^2{}_0R}{w}\int_0^{2\pi}\sin^2 xdx = \frac{I^2{}_0R}{w}\left(\frac{x}{2}\bigg|_0^{2\pi} - \frac{\sin 2x}{4}\bigg|_0^{2\pi}\right) = \frac{I^2{}_0RT}{2} \tag{6}$$

where i is the sinusoidal transient current, I_0 is the current amplitude, R is the internal resistance, T is the period, w is the current frequency, and dt denotes differentiation.

Therefore, the equivalent current of the sine wave is $I_{equal1} = \sqrt{\frac{I^2{}_0RT/2}{I^2{}_0RT}} = \frac{\sqrt{2}}{2}$.

To quantitatively demonstrate the reduction in energy consumption by adjusting the sinusoidal current waveform, we illustrate the case when $\eta = \frac{B_{Keep}}{B_{Amp}} = k$ is adjusted. At this point, the energy consumption in a single cycle is

$$i^2RT = 2R\left(\int_0^{\frac{T}{4}} I^2{}_0\sin^2 wtdt + \int_{\frac{T}{4}}^{\frac{T}{2}} k^2I^2{}_0dt\right) = I^2{}_0RT\left(\frac{1}{4}+\frac{k^2}{2}\right) \tag{7}$$

Thus, the equivalent current I_{equal2} of the proposed energy-saving waveform is

$$I_{equal2} = \sqrt{\frac{I^2{}_0RT(1/4+k^2/2)}{I^2{}_0RT}} = \sqrt{\frac{2k^2+1}{4}} \tag{8}$$

$$\lambda_{equal} = \frac{I_{equal2}}{I_{equal1}} = \sqrt{\frac{2k^2+1}{2}} \tag{9}$$

where λ_{equal} means the equivalent current ratio of energy-saving waveform and standard sinusoidal waveform with the same amplitude. Therefore, when $k = 0$, the ratio λ_{equal} is 70.7%; when $k = 0.2$, the ratio λ_{equal} is 73.49%.

In addition, when the amplitude of the waveform is the same, the energy consumption ratio of the energy-saving waveform and the sinusoidal waveform is

$$n = \frac{I^2{}_{equal2}}{I^2{}_{equal1}} = \frac{2k^2+1}{2} \tag{10}$$

when $k = 0.2$ in this work, the energy consumption of the energy-saving waveform is only 54% of that of the sinusoidal waveform.

Note S4. Influence of gripper tentacle on gripping effectiveness

We fabricated molds for sea anemone-inspired grippers with different tentacle lengths (L = 1, 3, 5, 7 mm) using 3D printing. A composite of 50 wt% magnetic silicone (NdFeB + Ecoflex 00-20) was cast into the molds to form the grippers (shell radius R = 15 mm), as shown in Fig. S12(a). Using a permanent magnet (N35, D = 50 mm, H = 20 mm), we tested the grippers' ability to grasp cylindrical objects of varying radii. When the tentacle length is relatively short ($L \leqslant 1$ mm), the gripper can only effectively grasp large objects ($R \geqslant 8$ mm) and performs poorly with smaller objects. As the tentacle length increases, the gripper's success rate in grasping smaller objects improves significantly, achieving a broader grasping range for objects with radii between 1 and 10 mm. However, when the tentacle length becomes too long (L = 7 mm or more), interference between the tentacles during the grasping process reduces the gripper's success rate. Based on these observations, we selected a tentacle length of L = 5 mm as the optimal design parameter for the gripper.

In addition, we investigated the effect of tentacle stiffness on grasping performance while keeping the tentacle geometry and distribution density constant. We fabricated grippers with four different tentacle materials: pure silicone (Ecoflex 00-20), magnetic soft composite (50 wt% magnetic powder, base Ecoflex 00-20), and rigid PLA (Fig. S12(c)). Using a permanent magnet, we tested the grippers' ability to grasp objects of three different masses (1.5 g, 3.0 g, 4.5 g) and measured the magnetic field threshold for switching during the grasping process. As shown in Fig. S12(d), increasing tentacle stiffness improves the gripper's success rate in grasping heavier objects. However, overly rigid tentacles (Gripper C) requires significantly higher magnetic field thresholds for actuation. The magnetic field threshold for Gripper C (48 mT) is 1.6 times that of Gripper B (30 mT). Therefore, using magnetic soft materials for the tentacles provides an optimal balance between performance and actuation efficiency, outperforming both pure soft and rigid materials.

Supplementary Figures:

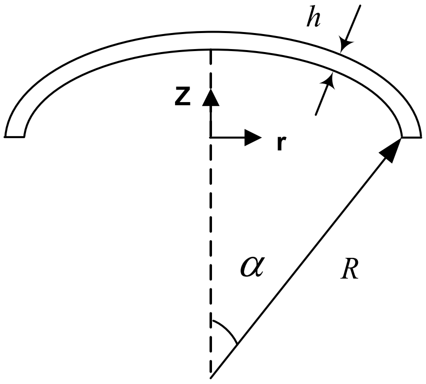

Fig. S1. Schematic structure of a dome-shaped shell. R means the curvature radius of the shell, α means the angle of a quarter of the shell, h means the thickness of the shell.

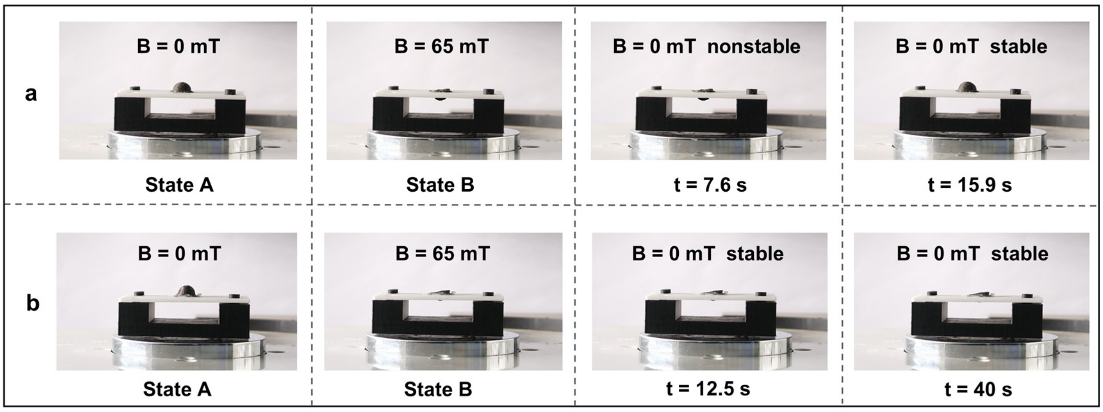

Fig. S2. Deformation processes of monostable and bistable shells. (a) Monostable shell (R = 5 mm, h = 1.5 mm). (b) Bistable shell (R = 5 mm, h = 0.5 mm).

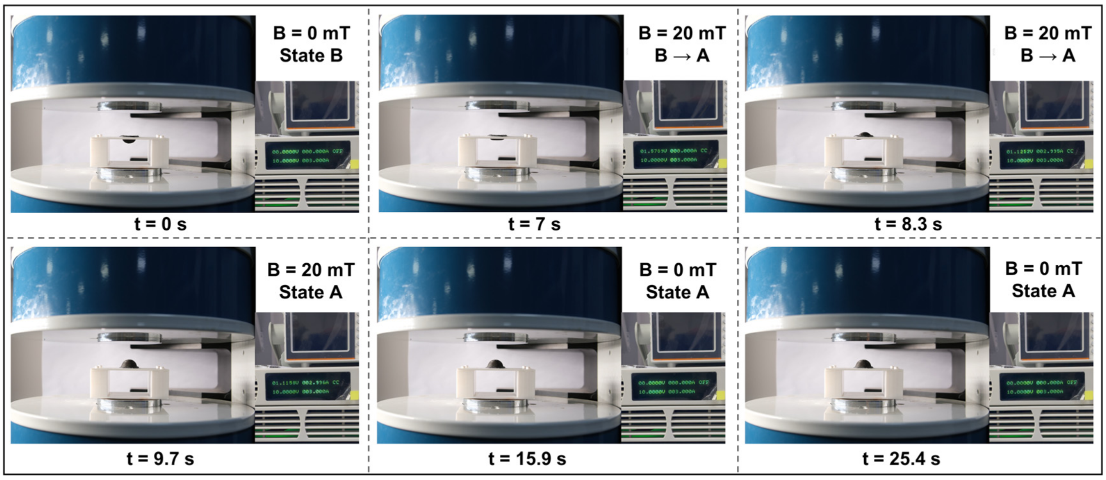

Fig. S3. Bistable dynamic process showing the transition from State B to State A.

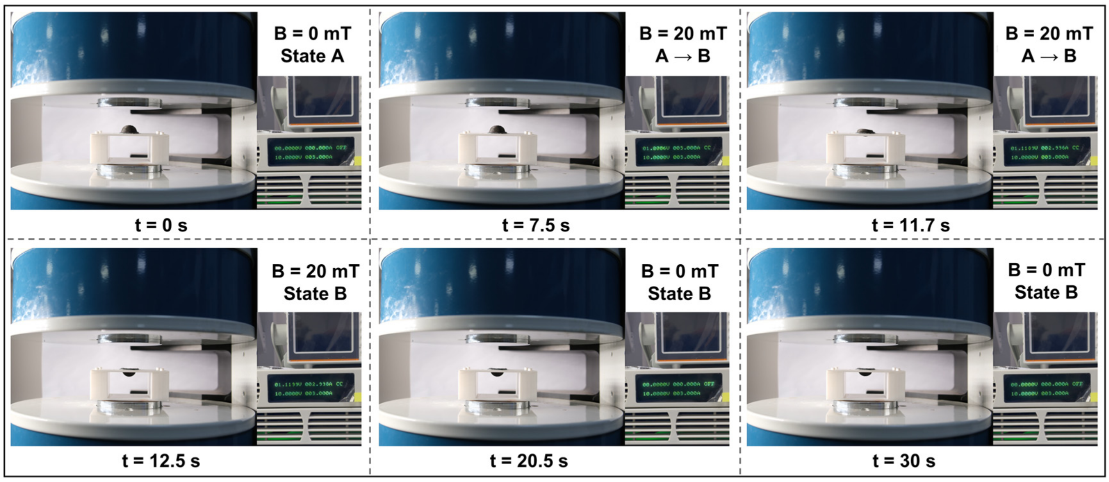

Fig. S4. Bistable dynamic process showing the transition from State A to State B.

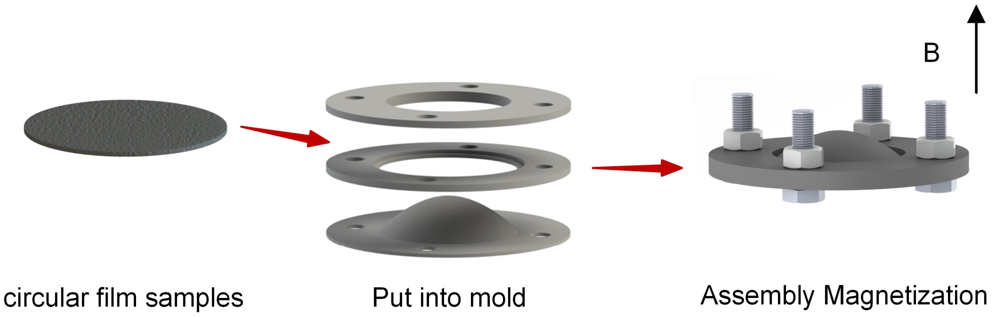

Fig. S5. Magnetization of circular magnetic soft samples by the hemispherical shell mold method. First, the samples were placed between two layers of circular fixing molds, and then they were pre-deformed by the top of the convex molds of the hemispherical shell, and the parts were fixed using epoxy screws. Under a pulsed magnetic field (> 2T), magnetization was performed, and then the screws were removed to obtain the samples magnetized by the mold method.

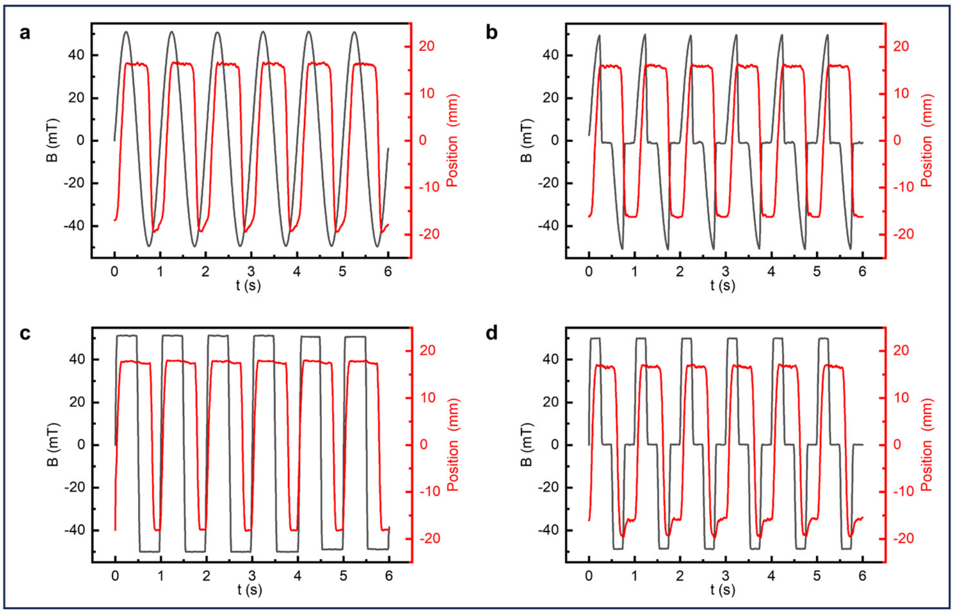

Fig. S6. Comparison of shape deformation curves under different actuation waveforms. **(a)** Sinusoidal waveform. **(b)** Energy-saving sinusoidal-like waveform (η = 0). **(c)** Square waveform. **(d)** Energy-saving square-like waveform. These waveforms can all achieve a deformation of 15 mm or more. Source data are provided as a Source Data file.

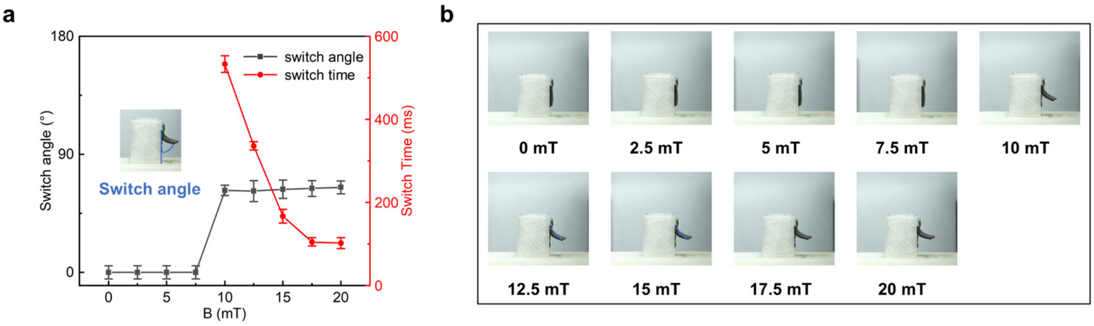

Fig. S7. Opening characteristics of the magnetic valve. **(a)** The relationship between the valve opening angle and the magnetic field strength, along with the variation in opening time (n = 3, data are presented as mean values +/− SD). Source data are provided as a Source Data file. **(b)** Optical experimental diagram showing the magnetic valve opening under different magnetic field strengths.

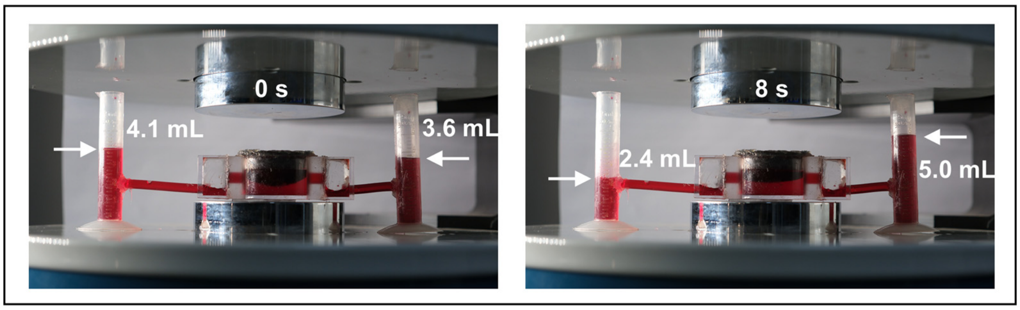

Fig. S8. Liquid pumping process under a 10 Hz, 20 mT sinusoidal magnetic field.

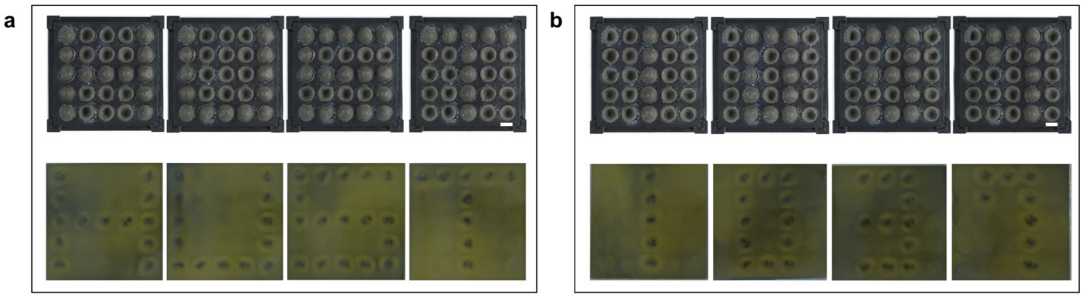

Fig. S9. Programming structure and display of the proposed metamaterial. (a) HUST display case (photography and magnetic display card observation). **(b)** 1037 display case (photography and magnetic display card observation).

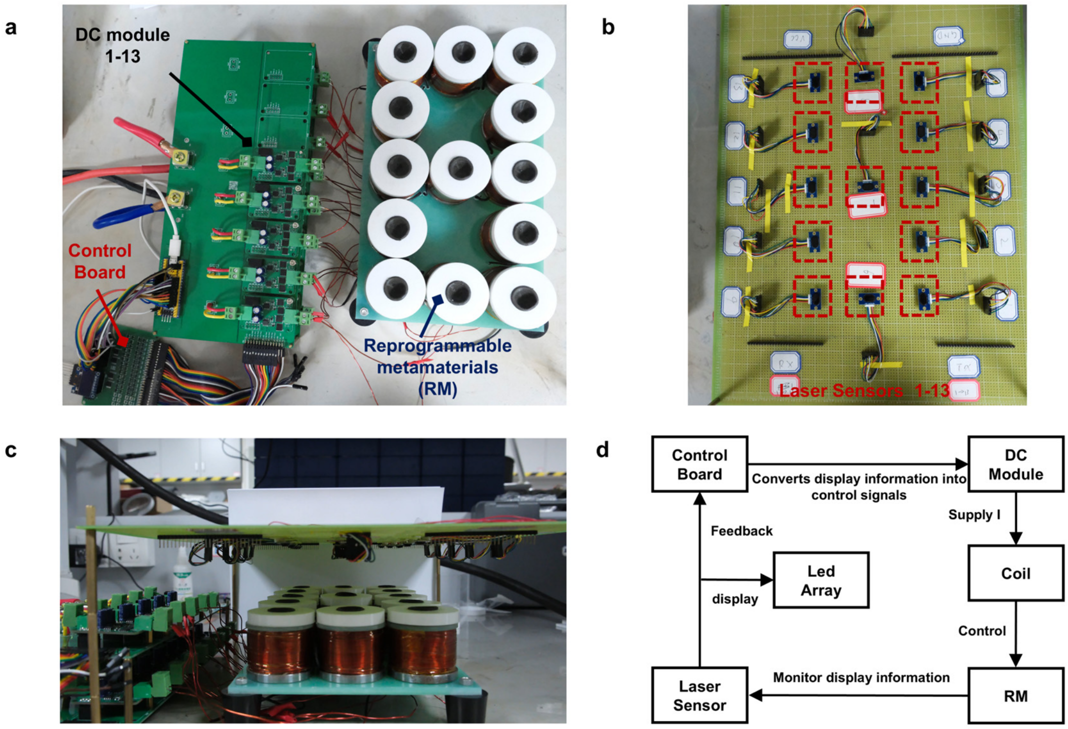

Fig. S10. Physical diagram and control strategy of the magnetic actuation system for reprogrammable metamaterials. (a) Diagram of components such as the power supply, control system, and array coils of the magnetic actuation system, where the DC bridge module regulates the magnitude and direction of the input current. **(b)** Experimental setup for the laser sensor detection module. **(c)** Overall diagram of the experimental system. **(d)** Logic diagram depicting the principle for detecting and refreshing the display. RM: Reprogrammable metamaterials.

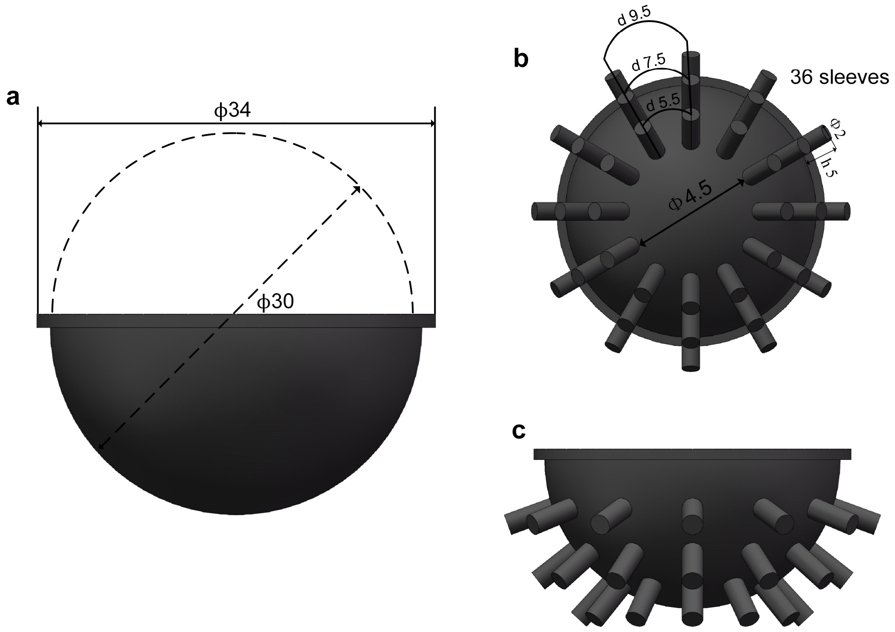

Fig. S11. Dimensions of the biomimetic sea anemone gripper. **(a)** Simplified display of the gripper diameter parameters. **(b)** Display of 36 cylindrical grasping elements, each with a diameter of 2 mm and a height of 5 mm, uniformly distributed around the circumference to facilitate grasping. **(c)** Schematic diagram of the full structure of the biomimetic sea anemone gripper.

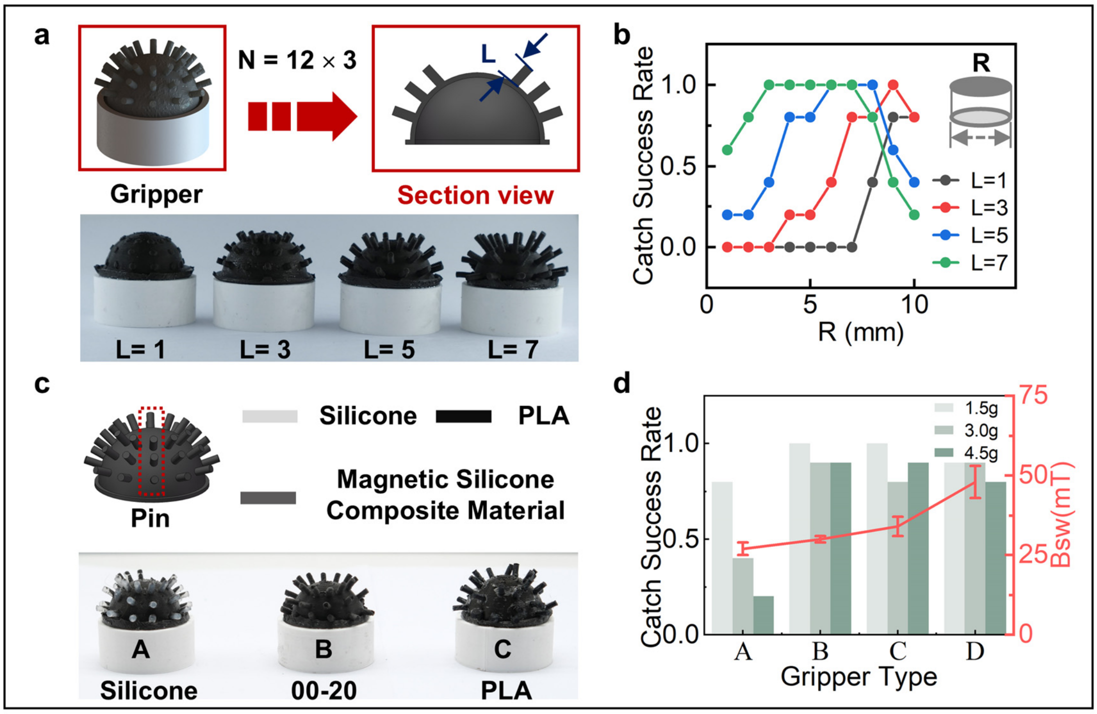

Fig. S12. Effects of tentacle length and stiffness on the grasping efficiency of the sea anemone-inspired gripper. (a) Experimental setup showing grippers with different tentacle lengths (*L*). **(b)** Grasping success rates of grippers with different tentacle lengths for cylindrical objects of varying radii (*R* = 1–10 mm, height *H* =10 mm). **(c)** Experimental setup showing

grippers with tentacles of varying stiffness. The tentacle materials of the three grippers are: (A) pure silicone, (B) magnetic silicone composite material (based on Excoflex 00-20), and (C) polylactic acid (PLA). **(d)** Grasping success rates of grippers with tentacles of varying stiffness for objects of different mass (1.5 g, 3.0 g, 4.5 g). (n = 3, data are presented as mean values +/− SD). Source data are provided as a Source Data file.

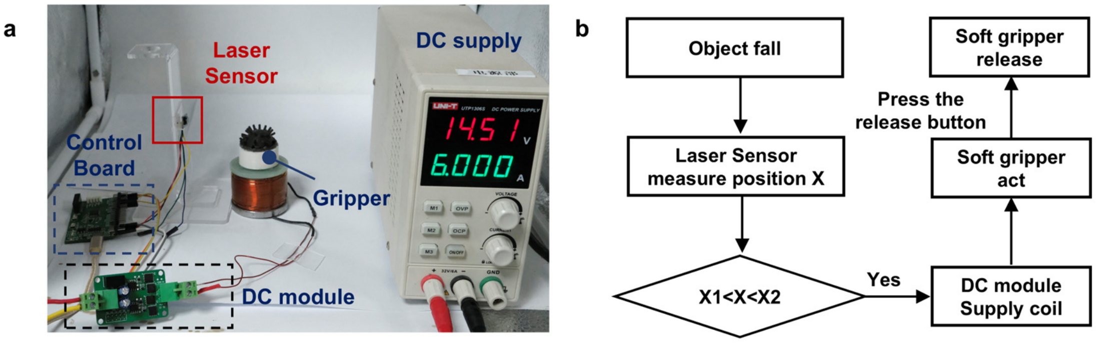

Fig. S13. Principle of the self-recognizing grasping anemone gripper. **(a)** Experimental setup for the self-recognizing grasping. **(b)** Logic diagram illustrating the recognition of grasping and releasing actions.

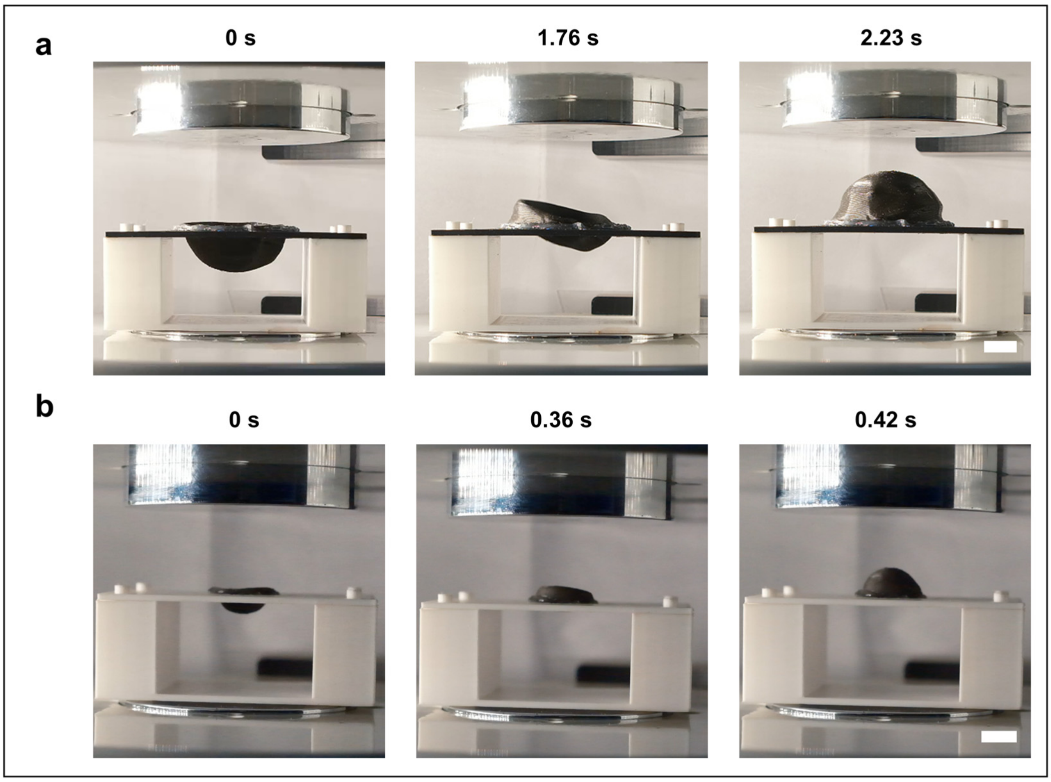

Fig. S14. Comparison of deformation processes of hemispherical shells with different sizes. (a) Deformation process of a magnetoactive soft hemispherical shell (R = 20 mm, H = 0.5 mm) under a static driving magnetic field of 10 mT. **(b)** Deformation process of a magnetoactive soft hemispherical shell (R = 10 mm, H = 0.5 mm) under a static driving magnetic field of 20 mT.

Table S1. Comparison of shape transformation characteristics of magnetoactive soft actuators.*

Reference	Size (mm)	Shape change (mm)	Shape change ratio	Magnetic field (mT)	Normalized shape change ratio (mT^{-1})
**[9]**	Φ 20×0.5 Folded	5.6	**0.280**	40 mT	**7×10^{-3}**
	Φ 20×0.5 Flat	2.17	**0.109**	40 mT	**2.71×10^{-3}**
**[10]**	Φ 16×1.4	1.0	**0.063**	89.9 mT	**7.01×10^{-4}**
**[11]**	Φ 20×1	3.40#	**0.170**	**300 mT**	**5.67×10^{-4}**
**[12]**	Φ 10×0.265	4.1	**0.410**	**938 mT**	**4.37×10^{-4}**
**[13]**	Φ 30×1.08	12.20##	**0.407**	**125 mT**	**3.25×10^{-3}**
**[14]**	Φ 30×0.5	7.30	**0.243**	**192 mT**	**1.27×10^{-3}**
**Our work**	Φ 40×1	35.77	0.894	40 mT	2.24×10^{-2}
	Φ 20×0.5	16.22	0.811	20 mT	4.06×10^{-2}
	Φ 40×0.5(1)	32.73	0.818	10 mT	8.18×10^{-2}

***:** To ensure a fair comparison, those soft actuators with fully edge-constrained configurations were considered in this table.

#: This value is not provided directly in the original article but is obtained through our estimation.

##: The article only reports the upward shape change. Based on the bistable symmetry of the spherical shell, we estimated the total shape change as twice the upward value.

(1): This data is obtained from Supplementary Fig S14.

Shape change ratio: A dimensionless value calculated by dividing the maximum deformation by a characteristic dimension (e.g., diameter or length).

Normalized shape change ratio: This is defined as the shape change ratio normalized by the applied magnetic field strength.

Bold font: This indicates that the shape change ratio is less than 0.5, the driving magnetic field exceeds 100 mT, and the normalized shape change ratio is below 2.24×10^{-2}.

Table S2. Performance comparison of magnetoactive soft diaphragm pumps.

Reference	Actuation Principle	Gradient-Free Actuation	Material type	Magnetic parameter	Normalized shape change ratio (mT^{-1})	Size (mm)	Average flow rate (mL/min)	Normalized flow rate (mL/min/mT)
**[15]**	Magnetic force	×	TPR (50A shore hardness) + Carbonyl iron particles	Coil: **443 mT, 0.5 Hz**	**$< 8\times10^{-5\#}$**	$40\times40\times18^{\#}$	**16.2**	**3.65×10^{-2}**
**[16]**	Magnetic force	×	PDMS + Carbonyl iron particles	Coil: **225mT, 1 Hz**	**2.1×10^{-3}**	30×10×0.7	**1.974**	**8.77×10^{-3}**
**[17]**	Magnetic force	×	Prepolymer (Neukasil RTV 23) + Iron particles	Coil: **430 mT, 1 Hz**	**$< 1.5\times10^{-3\#}$**	Φ12×320	78.5*	**0.18***
**[18]**	Magnetic torque	√	Flexible photosensitive resin (F39T) + NdFeB particles	Permanent magnet: periodic motion **0.14 Hz****	—	Φ44×1.8	**3.6**	—
**[9]**	Magnetic torque	√	Silicone Elastomer (Ecoflex00-20)+ NdFeB particles	Coil: 40 mT, 2Hz (Single diaphragm)	**$< 2\times10^{-3\#}$**	Φ20×0.5	**7.9**	**0.20**
				Coil: 20 mT, 5Hz (Double diaphragm)	**$< 3.5\times10^{-3\#}$**		**7.8**	**0.39**
**Our work**	Magnetic torque	√	Silicone Elastomer (Ecoflex00-20)+ NdFeB particles	Coil$^{(1)}$: 36.75 mT, 2Hz	6.8×10^{-3}	Φ40×1	22.72	0.62
				Coil$^{(1)}$: 36.75 mT, 5Hz			30.1	0.82

#: This value is not provided directly in the original article but is obtained through our estimation.

***:** This represents the maximum pumping flow rate when the pump body is operating. Since the article does not provide additional data, it cannot be converted into an average flow rate.

****:** The article does not provide the specific size of the permanent magnet but does specify its motion period (T = 7 s). Based on this information, we estimated the equivalent driving cycle for comparison purposes.

Coil[(1)]: For the energy-saving waveform with a frequency of 2Hz, a magnetic field of 50mT, and a duty cycle of 0.2 used in our work, the corresponding equivalent amplitude of a sinusoidal magnetic field with the same energy consumption is 36.75mT (**calculated using Eq. (9) in Note S2**).

TPR: Thermoplastic rubber materials. **NdFeB:** Neodymium-iron-boron. **PDMS:** polydimethylsiloxane.

Normalized shape change ratio: A dimensionless metric calculated by dividing the shape transformation amount by a characteristic dimension (e.g., diameter or length) and further normalizing it by the applied magnetic field strength.

Normalized flow rate: This is defined as the ratio of the average liquid pumping flow rate (except for Ref. 15, which reports the maximum flow rate) to the driving magnetic field strength.

Bold font: This indicates that the driving magnetic field must be a gradient field, the field strength is relatively high (> 100 mT), the actuation frequency is low (< 1 Hz), the shape change ratio per unit magnetic field is limited ($< 6.8 \times 10^{-3}\ mT^{-1}$), the flow rate is relatively low (< 22.72 mL/min), or the normalized flow rate is limited (< 0.62 mL/min/mT).

Table S3. Comparison of bistable dome-shaped soft actuators.

Reference	Actuation principle	Material type	Actuation parameter	Snap Time[3]	Size (mm)	Application case
**[19]**	Pneumatic loading	Dragon Skin + Ecoflex 30 + Smooth-Sil 950	10.2 kPa and 3.3 kPa[1]	**0.2 s**	Φ 20×3	■ Soft gripper<br>■ Soft earthworm
**[20]**	Temperature	Hydrogel	From 20 °C to 40 °C (PH=7)	**90 s**	Φ 30×2.2	—
	PH		From PH = 2 to 7 (20 °C)	**28 min**		
**[21]**	Magnetic force	PDMS + Carbonyl iron particles	**>200 mT[2]**	**0.14 s**	Φ 44×1.5	—
**[13]**	Magnetic torque and Pneumatic loading	Vinylpolysiloxane (VPS-32) + NdPrFeB particles	**125 mT**	—	Φ 30×1.08	■ Braille dot
**[14]**	Magnetic torque	PDMS + NdFeB particles	**192 mT**	**0.12 s**	Φ 30×0.5	■ Flytrap<br>■ Reconfigurable electronics<br>■ Dynamic bioreactor
**Our work**	Magnetic torque	Silicone Elastomer (Ecoflex00-20) + NdFeB particles	60 mT (f = 6 Hz)	67 ms	Φ 40×1	■ Soft Pump<br>■ Reprogrammable metamaterials<br>■ Bionic soft gripper
			40 mT (f = 6 Hz)	43 ms[4]	Φ 20×0.5	

1: The two sets of pressures correspond to the two stable states of the spherical shell.

2: Our estimation is based on the reported limiting switching distance of 14 mm from the permanent magnet, together with the magnetic field strength distribution diagram provided in the study.

3: It should be noted that when comparing different studies, snap time should be considered a reference metric rather than a strict benchmark, as it is influenced by various factors such as actuator dimensions, material stiffness, and testing conditions. To improve data comparability, we tried to adopt comparable test conditions (e.g., similar actuator dimensions) in this table.

4: This data is obtained from Supplementary Movie 16.

Bold font: This indicates that these bistable actuators in the literature have a relatively long response time (>0.1 s) or require a relatively large magnetic field (> 100 mT).

Supplementary References

[1] Zhao, J. *et al*. Smart adhesives via magnetic actuation. *Adv. Mater.* **34**, 2107748 (2022).

[2] Beltran-Huarac, J. *et al*. Magnetic control of protein expression via magneto-mechanical actuation of ND-PEGylated iron oxide nanocubes for cell therapy. *ACS Appl. Mater. Interfaces* **15**, 19877–19891 (2023).

[3] Chen, Y., Srinivasan, K., Choates, M., Cestarollo, L. & El-Ghazaly, A. Enhanced magnetic anisotropy for reprogrammable high-force-density microactuators. *Adv. Funct. Mater.* **34**, 2305502 (2024).

[4] Brinkmeyer, A., Santer, M., Pirrera, A. & Weaver, P. M. Pseudo-bistable self-actuated domes for morphing applications. *Int. J. Solids Struct.* **49**, 1077–1087 (2012).

[5] Brodland, G. W. & Cohen, H. Deflection and snapping of spherical caps. *Int. J. Solids Struct.* **23**, 1341–1356 (1987).

[6] Pauchard, L. & Rica, S. Contact and compression of elastic spherical shells: the physics of a ping-pong ball. *Philos. Mag. B* **78**, 225–233 (1998).

[7] Taffetani, M., Jiang, X., Holmes, D. P. & Vella, D. Static bistability of spherical caps. *Proc. R. Soc. A* **474**, 20170910 (2018).

[8] Skov, A. L. & Yu, L. Optimization techniques for improving the performance of silicone-based dielectric elastomers. *Adv. Eng. Mater.* **20**, 1700762 (2018).

[9] Lin, D., Yang, F., Gong, D. & Li, R. Bio-inspired magnetic-driven folded diaphragm for biomimetic robot. *Nat. Commun.* **14**, 163 (2023).

[10] Kim, Y. C., Lee, J. & Park, S. M. Optimization of magnetoactive polymer membranes using radial magnetization. *Mater. Today Commun.* **39**, 108705 (2024).

[11] Lin, D. *et al*. Magnetoactive soft drivers with radial-chain iron microparticles. *ACS Appl. Mater. Interfaces* **13**, 34935–34941 (2021).

[12] Chi, Y. *et al*. Magnetic kirigami dome metasheet with high deformability and stiffness for adaptive dynamic shape-shifting and multimodal manipulation. *Sci. Adv.* **10**, eadr8421 (2024).

[13] Abbasi, A., Chen, T., Aymon, B. F. G. & Reis, P. M. Leveraging the snap buckling of bistable magnetic shells to design a refreshable braille dot. *Adv. Mater. Technol.* **9**, 2301344 (2024).

[14] Chen, Z. *et al*. A magnet-driven soft bistable actuator. *Adv. Funct. Mater.* **34**, 2311498 (2024).

[15] Cao, X. *et al*. 3D printing ultraflexible magnetic actuators via screw extrusion method. *Adv. Sci.* **9**, 2200898 (2022).
[16] Cao, X., Xuan, S., Hu, T. & Gong, X. 3D printing-assistant method for magneto-active pulse pump: Experiment, simulation, and deformation theory. *Appl. Phys. Lett.* **117**, 241901 (2020).
[17] Fuhrer, R., Schumacher, C. M., Zeltner, M. & Stark, W. J. Soft iron/silicon composite tubes for magnetic peristaltic pumping: frequency-dependent pressure and volume flow. *Adv. Funct. Mater.* **23**, 3845–3849 (2013).
[18] Sun, H. *et al*. Magnetic field-assisted manufacturing of groove-structured flexible actuators with enhanced performance. *Addit. Manuf.* **80**, 103979 (2024).
[19] Rothemund, P. *et al*. A soft, bistable valve for autonomous control of soft actuators. *Sci. Robot.* **3**, eaar7986 (2018).
[20] Zhao, Q. *et al*. A bioinspired reversible snapping hydrogel assembly. *Mater. Horiz.* **3**, 422–428 (2016).
[21] Loukaides, E. G., Smoukov, S. K. & Seffen, K. A. Magnetic actuation and transition shapes of a bistable spherical cap. *Int. J. Smart Nano Mater.* **5**, 270–282 (2014).
