# Supplementary material for: Magnetoactive bistable soft actuators for programmable large shape transformations at low magnetic fields
Source: Nat Commun. 2025 Nov 5;16:9714. doi: 10.1038/s41467-025-64855-4 (PMC12589526; doi:10.1038/s41467-025-64855-4)
Supplement: Supplementary file 2 — Description of Additional Supplementary Files [file 41467_2025_64855_MOESM2_ESM.pdf]

Description of Addifional Supplementary Files

Supplementary Movie 1: Deformation processes of monostable and bistable shells.

Supplementary Movie 2: Bistable switching process with different initial configurations.

Supplementary Movie 3: Magnetoactive bistable soft actuator’s 100-cycle operating process.

Supplementary Movie 4: Switching process under a static magnetic field.

Supplementary Movie 5: Switching process under a sinusoidal magnetic field.

Supplementary Movie 6: Magnetoactive soft pump inflates four 16-inch HUST letter balloons in sequence.

Supplementary Movie 7: Magnetoactive soft pump for stable delivery of Ponceau solution.

Supplementary Movie 8: Liquid pumping process under a high-frequency sinusoidal magnetic field.

Supplementary Movie 9: Magnetoactive soft pump for liquids with different viscosities.

Supplementary Movie 10: Closed-loop precision pumping for water in magnetoactive soft pumps.

Supplementary Movie 11: Process of writing and erasing in the permanent magnet actuation system.

Supplementary Movie 12: Writing and displaying the information “1037” and “HUST” in the permanent magnet actuation system.

Supplementary Movie 13: 9s digital countdown display in the electromagnetic actuation systems.

Supplementary Movie 14: Gripping process of a 3D bionic anemone soft gripper.

Supplementary Movie 15: Self-recognizing and self-grasping bionic anemone soft gripper.

Supplementary Movie 16: Switching process of a magnetoactive bistable soft actuator (Φ20mm×0.5mm) under different magnetic fields.
